# Injection of seminal fluid into the hemocoel of honey bee queens (*Apis mellifera*) can stimulate post-mating changes

**DOI:** 10.1038/s41598-020-68437-w

**Published:** 2020-07-20

**Authors:** W. Cameron Jasper, Laura M. Brutscher, Christina M. Grozinger, Elina L. Niño

**Affiliations:** 1 Department of Entomology and Nematology, University of California Davis, One Shields Ave, Davis, CA 95616 USA; 2 Department of Entomology, Center for Pollinator Research, Huck Institutes of the Life Sciences, Pennsylvania State University, University Park, PA 16802 USA

**Keywords:** Reproductive biology, Transcriptomics

## Abstract

Honey bee queens undergo dramatic behavioral (e.g., reduced sexual receptivity), physiological (e.g., ovary activation, ovulation, and modulation of pheromone production) and transcriptional changes after they complete mating. To elucidate how queen post-mating changes are influenced by seminal fluid, the non-spermatozoa-containing component of semen, we injected queens with semen or seminal fluid alone. We assessed queen sexual receptivity (as measured by likelihood to take mating flights), ovary activation, worker retinue response (which is influenced by queen pheromone production), and transcriptional changes in queen abdominal fat body and brain tissues. Injection with either seminal fluid or semen resulted in decreased sexual receptivity, increased attractiveness of queens to workers, and altered expression of several genes that are also regulated by natural mating in queens. The post-mating and transcriptional changes of queens receiving seminal fluid were not significantly different from queens injected with semen, suggesting that components in seminal fluid, such as seminal fluid proteins, are largely responsible for stimulating post-mating changes in queens.

## Introduction

Managed honey bee (*Apis mellifera*) colonies provide an estimated $175 billion in pollination services for a large variety of crops worldwide with an estimated $14.6 billion value in the United States alone^1–3^. While the number of honey bee colonies has increased between 1961 and 2007^4^, the U.S. and some regions of Europe have exhibited high annual colony losses, with the U.S. experiencing an average annual loss of 33% recorded since 2006^5–13^. Premature queen loss is one of the top causes of colony loss reported by beekeepers both in the U.S. and in Europe^6,7,13^. Therefore, improving colony health and survivorship in large part will depend on understanding factors affecting queen health and productivity as well as investigating the role of drones (haploid male bees) on queen health.

A honey bee colony consists of a single egg-laying queen, hundreds to thousands of seasonal drones, and tens of thousands of sterile female workers^14^. Approximately 1 week after emergence as an adult, the queen will take one or more mating flights to large congregations of males called drone congregation areas (DCAs)^14,15^. Honey bee queens are polyandrous, which means they mate with multiple drones; queens have recently been reported to mate as many as 34–77 drones^16^. During copulation, drone semen is transferred to the queen’s oviduct where only 3–5% of each drone’s spermatozoa are stored in a specialized organ called the spermatheca^14,15,17,18^.

The process of mating initiates dramatic behavioral, physiological, and molecular changes in queens^19–24^. Behaviorally, mated queens become sexually unreceptive, as evidenced by the cessation of mating flights and their reduced attraction to light^14,25^. The ovaries of newly-mated queens begin activating and their ovarioles thicken and start to produce eggs, upon which the queens initiate egg-laying and continue to do so for the rest of their lives^26,27^. The amount and composition of pheromones the queen produces also changes after mating^21,28–36^. This results in greater elicitation of worker retinue response, which consists of licking and antennating the queen to distribute her pheromones throughout the colony^21,28–36^.

Investigations of molecular changes during the honey bee mating process have primarily focused on the transcriptional profiles of the brains, ovaries, and the fat bodies of queens that are instrumentally inseminated, naturally-mated, exposed to carbon dioxide (CO_2_), or both physically manipulated and exposed to CO_2_ as compared to virgin queens^37–42^. As such, the drastic behavioral changes queens exhibit after mating have been associated with regulation of vision-, immunity-, olfaction-, and metabolism-associated genes in the brain^37–40,42^. Virgin queens treated with CO_2_ resemble naturally mated queens in their post-mating behavioral (termination of mating flights) changes and brain transcriptional profiles^37,40,43,44^. Additionally, exposure to CO_2_ with or without physical manipulation of the vaginal tract promotes ovary activation and egg-laying behavior^40,43^. Queen behavior is also affected by the instrumental insemination process itself, where insemination with saline solution at a high volume or co-treatment of CO_2_ and physical manipulation promote termination of mating flights and ovary activation^40,41^.

Molecules in seminal fluid, a component of semen that does not contain spermatozoa, also likely influence post-mating changes in honey bee queens. The influence of seminal fluid and seminal fluid proteins (SFPs) on female fertility and behavior has been extensively studied in *Drosophila* and increasingly studied and supported in other insects, including mosquitos, crickets, ants, moths, and beetles^45–74^. In *Drosophila*, seminal proteins alone can initiate post-mating changes in females^46,60,64,70,72,75^. As is the case with other insects, the honey bee drone seminal fluid proteome contains multiple SFPs related to defense, energy production, metabolism, and signal transduction^76^. However, the mechanistic roles that drone SFPs have on post-mating changes specifically in honey bee queens are under-studied. While several prior studies^33,39,41,77^ have begun to reveal the impact of semen on queen mating and reproductive changes, they either did not attempt to isolate the effect of seminal fluid alone^39^ or did not attempt to fully remove the potential effects of anesthetizing agent and physical manipulation on the queen genital tract^42^, which have been shown to largely drive queen changes similar to those post-mating^40^.

**Figure 1:**
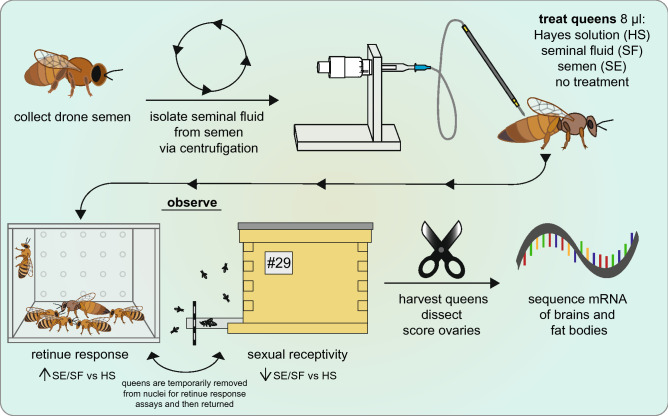
Procedure for queen experiments. Semen was first collected from ~ 300 drones. In order to isolate seminal fluid from semen, semen was centrifuged to pellet sperm. The seminal fluid-containing supernatant was then collected. Queens were injected between the second and third abdominal tergite with 8 µL Hayes solution (the semen diluent used), seminal fluid, or semen. A subset was left untreated (virgins). Queens were observed for mating flight attempts in small mating nuclei as an assay for sexual receptivity. A subset of those queens was taken from the mating nuclei and placed into a Plexiglass container where the number and frequency of workers performing retinue response to the queen were recorded. After the observation period ended, queens were harvested and dissected. Ovaries were scored for development and fat bodies and brains were collected for RNA extraction and RNAseq analysis. Parts of the figure were adapted with permission from Brutscher et al., Insects, 2019 under the Creative Commons Attribution 4.0 International Public License (https://creativecommons.org/licenses/by/4.0/legalcode).

In this study, we sought to further uncouple the effect of molecules in seminal fluid versus those associated directly with spermatozoa on behavior, physiological changes including pheromone production and both brain and fat body gene expression in queens (Fig. 1). Since our prior work clearly demonstrates there are confounding effects from CO_2_ narcosis and genital tract manipulation^40,43^, we opted to inject instead of instrumentally inseminate our experimental queens with semen or seminal fluid. While the biological relevance of this method in honey bees it is not yet known—it has yet to be determined if seminal fluid components naturally exit the reproductive tract of honey bee queens—injecting seminal fluid components into the hemocoel is a well-established method for examining post-mating changes in many other insects^78–87^. We then examined queen sexual receptivity (as measured by mating flight attempts, as the only time a young queen leaves the colony is when she is sexually receptive), queen ovary activation, gene expression patterns in the brain and fat body, and worker retinue response around experimental queens. We chose to examine gene expression in the brain in order to compare our results with other studies examining the effect of mating factors on queen brain gene expression^37–40,42^. The fat bodies were also examined because they are the primary energy storage and metabolic organ in the insect body (reviewed in^88^). After the queen mates, her fat body tissue permanently increases production of vitellogenin proteins as well as other nutrients to aid egg development^89^. Thus, it is important to understand the effects of seminal fluid or semen on fat body expression.

Findings of our study suggest that hemocoel injection of molecules contained in seminal fluid, such as proteins, lipids, or sugars, are largely responsible for stimulating post-mating changes in queens. The results provide an important foundation for future studies that will elucidate complex factors and processes driving post-mating changes in a social insect, and from a practical perspective, could contribute towards reducing queen and drone-related colony losses.

## Materials and methods

### General queen rearing

The field poriton of the study was performed during June–August, 2013. Honey bee queens (*Apis mellifera carnica*) were reared at Pennsylvania State University (University Park, PA). The source colony used for queen grafting was headed by a single-drone inseminated queen (SDI; Honey Bee Insemination Services, Coupeville, WA). Young female larvae (~ 24 h-old) were grafted into commercially available queen cups (Brushy Mountain Bee Farm, Moravian Falls, NC) with royal jelly and placed in queenless colonies to be reared into queens by workers^90^. Since honey bees exhibit haplodiploidy, the queens used in this study were highly related with the average coefficient of relatedness (G) of 0.75. A week after grafting, sealed queen cells were placed in an incubator (32.5 °C and ~ 50% RH) where they remained for 48 h so as to prevent workers from tearing down the queen cells. Two days prior to their expected emergence, queen cells were placed into individual miniature mating nuclei with approximately 1,000 workers (*Apis mellifera ligustica* origin), one frame of young brood, one frame of honey and pollen, and one empty frame^21,30,38,40^.

### Preparation of injection material and queen injections

Hayes solution (0.15 M NaCl, 1.80 mM CaCl_2_, 2.68 mM KCl, 1.19 mM NaHCO3, adjusted to pH 8.7 using NaOH) was prepared as in Baer et al. (2009)^76^. One day before the injections (6 days after queen emergence), semen was collected from approximately 200 mature drones from three unrelated *Apis mellifera ligustica* colonies per standard instrumental insemination practices. As per standard instrumental insemination practices, semen was stored overnight at room temperature^91^.

On the injection day, seminal fluid was isolated from an aliquot of the semen as in Baer et al. (2009)^76^ except the semen was diluted with Hayes solution at a 1:1 ratio and centrifuged (25 min at 3,000 rpm) at room temperature. Centrifugation pelleted the sperm, while the supernatant contained seminal fluid and diluent. The seminal fluid-containing supernatant was then pipetted into a new tube. All queens were then marked on their thoraces with a marking pen (Dadant and Sons, Inc., Hamilton, IL), and their left wing was clipped to prevent mating flights. Queens were randomly assigned into one of four groups: Hayes solution-injected queens (HS, n = 13), semen-injected queens (SE, n = 16), seminal-fluid injected queens (SF, n = 15), and non-injected virgin control queens (V, n = 13). We did not examine naturally mated queens due to the complications of controlling the timing and frequency in which they would partake in mating flights and complete their mating cycles. To minimize confounding effects from the time of day in which the treatments were received, queens were collected from the mating nuclei in batches, each containing an equal distribution of each treatment group, throughout the day.

Queens were injected with 8 µL of their respective solution directly into the hemocoel (similar to what has been performed in many other insects^78–87^, but not previously performed on honey bee queens) between the second and third abdominal tergite with a pulled glass needle attached to the insemination instrument (Schley Compact Model II Instrument; Honey Bee Insemination Services, Davis, CA, US). We chose to inject queens with an 8 µL aliquot since this is the volume typically used during instrumental insemination^91^. No anaesthetic was used for injections since CO_2_ causes changes (e.g., ovary activation) in queens similar to those after mating^40,43,92,93^, and the effects of other anaesthetics including chilling have not been studied in detail. Although it appears that chilling does not have the same stimulatory effect of CO_2_, we cannot yet speak to any other potential negative effects (Brutscher, Niño unpublished data). After the treatment, queens were returned to their respective mating nuclei.

### Mating flight behavior

Mating nuclei were equipped with clear Plexiglas runways with queen excluder entrances^38^. This allowed the observer to be able to determine if the queen was attempting to take a mating flight (she would enter the runway and remain there for some time before returning to the colony), but prevent the queen from actually taking a flight. Behavioral observations started the day after the treatment (eight days post-emergence). Since queens take mating flights in the afternoon, each hive was observed every day for four days between 1–6 pm in ten-minute intervals for attempted mating flights. Only the queens surviving until the end of the experiment were used for statistical analyses. Chi-square analysis was performed in R^94^ to determine differences in the number of queens attempting mating flights or laying eggs among the three groups.

### Measurement of queen attractiveness to workers (retinue response)

Five days after the injections, a randomly selected subset of queens from each of the four treatment groups were collected and introduced into Plexiglas cages containing 30 seven-day-old workers. To obtain the workers for the retinue assay, frames of emerging brood (*Apis mellifera carnica*) were taken from one colony headed by a SDI queen (Honey Bee Insemination Services, Coupeville, WA) to control for variance in worker retinue response due to genetic variability^39^. Brood frames were placed in an incubator at 34.5 °C and 50% relative humidity until emergence. Groups of 30 one-day-old workers were placed in individual Plexiglas cages (10 × 100 × 7cm).

Cages were equipped with a patty of MegaBee pollen supplement (S.A.F.E. R&D, Tuscon, AZ), one 1.5 ml microcentrifuge tube with 50% sucrose–water solution, and one 1.5 ml microcentrifuge tube with water. Since worker adult behavioural maturation depends on the presence of queen pheromone^95^, we reared workers in the presence of 0.1 queen equivalent (Qeq) of synthetic QMP (Pherotech, Canada) which was placed on a glass cover slip and allowed to evaporate before it was introduced into a cage^30,77^. Synthetic QMP was replaced daily at approximately the same time of the day, and sucrose and water were replaced every other day. Cages were kept in an incubator at 34.5 °C and 50% RH for 7 days. At this time, queens were introduced into individual cages and allowed 1 h to adjust to the new environment. After the acclimation period, the number of workers antennating and licking the queen (retinue response) was recorded (Supplemental Table S1). Observations were repeated every 5 min over a 35 min period for a total of 8 observations. After the observations were completed, queens were returned back into their respective nuclei. They were collected on dry ice in the afternoon of the following day and transferred to a − 80 °C freezer until further processing. Retinue response data was log-transformed and a two-way repeated measures ANOVA followed by post-hoc pairwise t-tests with Bonferroni multiple testing correction was performed in R to evaluate the effect of the different treatments over time on worker retinue response.

### Tissue dissection and ovary evaluation

The head and thorax of individual queens were detached from the queen abdomen and stored at − 80 °C. Later, the queen heads were partially lyophilized and brains were dissected out on dry ice and preserved in RNAlater at − 80 °C until gene expression analysis. Queen abdomens were dissected on ice and in cold RNAlater solution (Qiagen, Valencia, CA). Eviscerated abdominal cuticles lined with fat body tissue were placed into individual Eppendorf tubes with RNAlater and stored at − 80 °C for gene expression analysis.

Spermathecae were examined to confirm that none of the queens were mated (a clear spermatheca indicates a virgin queen, while mated queens have spermathecae that appear white and opaque to tan and “marbled”). The level of ovary activation was determined for individual queens by assigning a score of 1 through 4 (1 = no development; 2 = larger ovaries, but without easily discernible ovarioles; 3 = easily discernible ovarioles, but no eggs; 4 = complete development, mature eggs present^38^ (Supplemental Table S1). To determine any differences in queen ovary activation among the groups, the Kruskal–Wallis Rank Sum test was applied to the data using the JMP 7.0 software (SAS, Cary, NC).

### RNA isolation

Abdominal cuticles and brain tissues were removed from the − 80 °C freezer and placed in 2 ml tubes containing RNAse-free beads and Qiazol (QIAGEN). Tissues were immediately homogenized using the Benchmark BeadBlaster at max speed (Benchmark Scientific, Edison, NJ). Brain tissues were homogenized for 30 s and abdominal cuticles were homogenized for 45 s, and both were chilled on ice for 30 s. RNA was then extracted using the standard methods and kit contents of the QIAGEN RNeasy RNA Extraction kit. RNA integrity^96^ was checked using the Experion Automated Electrophoresis System.

### Library preparation and sequencing

Libraries were prepared using the standard protocols and kit contents of the NEBNext Ultra RNA Library Prep Kit for Illumina. Concentration of input RNA was standardized for all samples. Libraries were sequenced on the Illumina HiSeq 2,500 (100 bp single-end) at the Vincent J. Coates Genomics Sequencing Laboratory at the University of California, Berkeley. Thirty-eight samples were distributed across six lanes and maximally diversified by tissue type, treatment, and batch. The raw reads are available at the GEO repository (GSE145395). An average of 29.7 million reads per sample were obtained with standard deviation of 5.8 million. Total read counts for each library are available in Supplemental Table S1.

### RNA-Seq analysis

Reads were filtered for quality using the cutadapt software package version 1.8.3^97^. Adapter contamination was removed and a minimum average quality score of 25 was required. The program FastQC was used to confirm that the resultant libraries maintained good sequence quality (> Q30) and that all adaptors were removed. Using FastQC^98^, it was also determined that many of the samples had redundant sequences, which were queried against the NCBI Nucleotide collection (nr/nt) using blastn^99^, for which they were identified as belonging to the Deformed Wing Virus (DWV) genome (GCA_000852585.1). Filtered reads were then aligned to the most recent build (Amel_HAv3.1) of the *Apis mellifera* genome^100^ and the Deformed Wing Virus genome (GCA_000852585.1) using Hisat2 version 2.1.0^101^ under default parameters. Reads were also aligned to the HoloBee Database v2016.1, a curated FASTA containing genomic sequences for honey bee-associated microbes and pathogens (https://data.nal.usda.gov/dataset/holobee-database-v20161), using Hisat2 version 2.1.0^101^ under default parameters to ensure no significant number of reads aligned to any pathogen genomes other than DWV. The average number of reads aligning to the *Apis mellifera* genome was 73% in brain samples and 52% in fat body samples (Supplemental Table S1). DWV was the most prevalent and abundant virus throughout all samples; the average percent of reads aligning to the DWV genome was 19% in brain samples and 41% in fat body samples (Supplemental Table S1). A Kruskal–Wallis test was performed to determine if DWV alignment rates were associated with treatment in R.

Using reads from samples that aligned to the *Apis mellifera* genome, gene read counts were generated using the HTSeq software version 0.9.1^102^ with the -i Dbxref and -t gene options. Differential gene expression was assessed using the limma software package^103^ using default parameters (Benjamini–Hochberg correction; FDR < 0.05). Briefly, genes that had fewer than 20 aligned reads in each sample were filtered from analysis (min.count = 20). Read counts were then normalized via the default trimmed mean of M-values (TMM) method. Pairwise comparisons of gene expression were conducted between each treatment group (i.e., virgin queens (V), Hayes solution (HS), seminal fluid (SF), and semen (SE)) within each tissue type (i.e., brain and fat body) for a total of 12 comparisons (Table 1 and Supplemental Tables S2–S5). DEGs with Benjamini-Hochberg (BH) adjusted p-values < 0.05 were considered statistically significant. Multi-dimensional scaling (MDS) plots representing Euclidean distances between each sample was generated in Limma.

**Table 1:** Number of differentially expressed genes (FDR < 0.05) between pairwise comparisons of fat bodies and queens from three treatments: Hayes solution-injected queens (HS), queens injected with seminal fluid (SF), and queens injected with semen (SE).

	SF vs HS	SE vs HS	SE vs SF
**Fat body**			
UP	65	194	0
Down	66	148	0
**Brain**			
Up	404	54	194
Down	272	60	40

Upon initial differential expression analysis, there were no significant differences in gene expression in any of the comparisons in the brain. MDS analysis was performed to determine if the fat body and brain samples clustered by treatment. Data from retinue response, mating flight behavior, and DWV alignment rates were overlaid with the MDS plot, after which, it appeared that samples with DWV alignment rates < 1% (i.e., most virgin samples, one seminal fluid brain sample, and one semen brain sample) largely clustered together in both tissue types (Supplemental Fig. S1). There was no statistically significant difference in DWV alignment rates between the HS and SF and SE queens in the fat bodies or the brain (Kruskal Wallis; chi-squared = 19, df = 17, p-value = 0.3285; Kruskal–Wallis chi-squared = 16.699, df = 16, p-value = 0.4053). It does not appear that injection substance affected DWV infection status or that DWV infection status would greatly affect differential gene expression results between injection treatment groups. The majority of virgin queens, however, had little to no detectable reads aligning to the DWV genome, so injection regardless of substance may have made queens more susceptible to DWV infection^104^. Previous research has shown that infection with DWV causes changes in honey bee gene expression^105–109^. Thus, an additional differential expression analysis excluding samples with DWV alignment rates < 1% was performed. In this sample subset, there still was no statistically significant difference in DWV alignment rates between the HS and SF and SE queens in the fat bodies or the brain (Kruskal Wallis; chi-squared = 11.2, df = 13, p-value = 0.5941; Kruskal–Wallis chi-squared = 10, df = 10, p-value = 0.4405). The results from this revised RNAseq analysis are what will be presented and assessed in the results and discussion.

In order to visually compare DEGs lists in each treatment, Venn diagrams were created^110^. Venn diagram analysis was also performed to identify which DEGs from this study were also regulated in prior microarray and RNAseq studies examining queen post-mating changes^37–42^. Instead of NCBI gene IDs, available BeeBase (Amel_HAv3.1) accession numbers were used as common gene identifier for these analyses (Supplemental Tables S6–S8).

### Gene ontology

To further investigate the function of the DEGs that were regulated between SF and SE queens versus HS queens, OrthoDB v10.1 OGid identifiers were used to determine orthologs between the *Drosophila melanogaster* genome (GCA_000001215.4 Release 6 plus ISO1 MT) and the *Apis mellifera* genome (Amel_HAv3.1) because there is a greater amount of gene ontology information for *D. melanogaster* genes compared to *Apis mellifera*. OrthoDB determines orthology between species with a clustering of best-reciprocal-hits method^111^. There were 7,665 honey bee genes that were identified as orthologs to genes encoded by the fruit fly *D. melanogaster* genome (Supplemental Table S9). Of the 8,201 gene transcripts detected in the fat body samples, *D. melanogaster* orthologs were identified for 6,178 genes. Of the 9,030 gene transcripts detected in the brain samples, *D. melanogaster* orthologs were identified for 6,556 genes. DAVID 6.8^112^ was used to identify biological process, molecular function, and cellular component gene ontologies (-FAT classification level) of the available *D. melanogaster* orthologs of the DEGs from the following honey bee pairwise comparisons: fat body SF vs HS, fat body SE vs HS, brain SF vs HS, and brain SE vs HS (DEGs, their respective fly orthologs, and GO annotations are available in Supplemental Tables S2–S5). The term FAT represents a level of GO term specificity. DAVID’s classification system provides GO lists labeled from 1 to 5 (e.g., GOTERM_BP_1), with increasing specificity. DAVID 6.8 was also utilized to perform functional enrichment analysis, which consists of a modified Fisher’s exact test that produces an EASE score, on the individual DEG lists. The 6,178 *D. melanogaster* orthologs identified for the gene transcripts in the fat body transcriptomes (Supplemental Table S9) were used as the background for analysis of the fat body DEG lists and the 6,556 genes *D. melanogaster* orthologs identified for the gene transcripts in brain transcriptomes (Supplemental Table S9) were used as the background for analysis of the brain DEG lists. Gene ontology enrichment clusters were considered statistically significantly enriched if the EASE score was p < 0.05 after BH correction for multiple testing.

### Quantitative PCR (qPCR) validation of RNAseq results

Quantitative PCR was used to validate RNAseq differential expression results of four genes (i.e., *serine protease snake*, *LOC408643*, *antitrypsin*, and *LOC409674*) in fat body samples of Hayes-, seminal fluid-, and semen-injected queens. According to the RNAseq results, these four genes were observed to be upregulated in the fat bodies of seminal fluid- and semen-injected queens as compared to Hayes-injected queens. All qPCR reactions were performed in triplicate using 2 μL of cDNA as template. Each 10 μl reaction was composed of cDNA template, Thermo Scientific Maxima SYBR Green qPCR Master Mix (2X), and forward and reverse primers (600 nM each). No template and no RT enzyme negative controls were used in all qPCR analyses. A CFX384 Touch Real-Time PCR Detection instrument (BioRad) was used, and the thermocycler conditions included a pre-incubation at 95 °C (10 min) and 40 cycles of 95 °C (15 s), 55–60 °C (30 s), 72 °C (30 s), and a final elongation 72 °C (4 min). Primers were designed using Primer3Plus (Supplemental Table S10). The ribosomal protein 8, *Am rpl8*, was used as the housekeeping gene for qPCR^113^. Melt point analysis and 2% agarose gel electrophoresis was used to confirm qPCR specificity. The efficiency of each primer was calculated using qPCR assays of cDNA dilution series and plotting the log10 of the concentration versus the crossing point threshold (C(t)) values and using the primer efficiency equation, (10(1/Slope) − 1) × 100) (Supplemental Table S11). The fold change for each target gene (TG) was calculated using the ΔΔC(t) method in which ΔC(t) = TG C(t) − rpl8 C(t), and ΔΔC(t) = sample ΔC(t) − average Hayes-injected ΔC(t).

## Results

### Mating flight behavior

**Figure 2:**
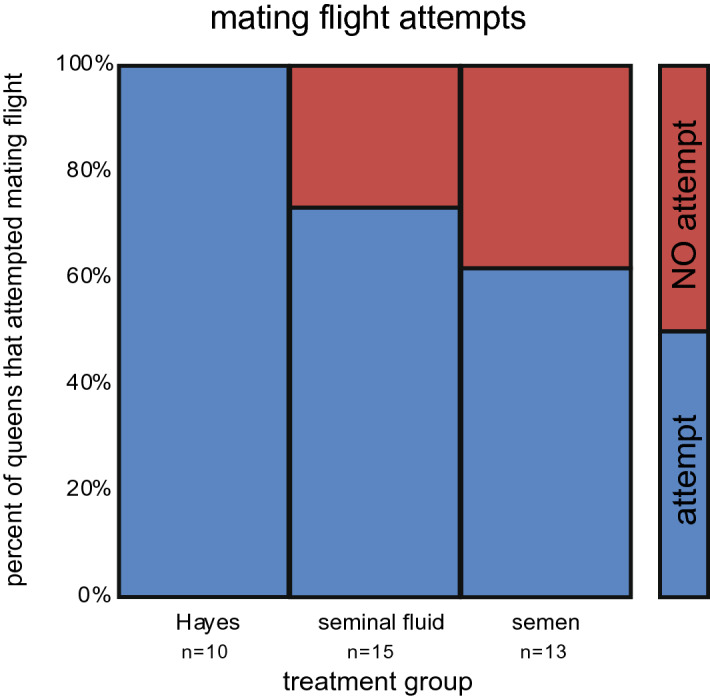
Queen Mating Flight Attempts (sexual receptivity). There was a significant difference in the percentage of queens from the different treatments attempting mating flights (chi-square (Pearson) = 10.526, df = 1, p < 0.005; Fig. 2). While 100% of HS queens attempted mating flights during the observation period, only 62% of SF queens and 73% of SE queens attempted mating flights. Blue represents queens that attempted a mating flight and red represents queens that did not attempt a mating flight.

We assessed common proxies of queen quality and health in order to compare queens injected with either seminal fluid (SF) or semen (SE) to queens injected with Hayes solution (HS). Cessation of mating flights or sexual receptivity can be used as a behavioral proxy to determine if a queen has undergone changes associated with completion of mating. There was a significant difference in the percentage of queens from the different treatments attempting mating flights (chi-square (Pearson) = 10.526, df = 1, p < 0.005; Fig. 2, non-injected virgin control data is shown in Supplemental Fig. S2). While 100% of HS queens attempted mating flights during the observation period, only 62% and 73% of SF and SE queens attempted mating flights, respectively. There were no significant behavioral differences observed between SF and SE queens.

### Measurement of queen attractiveness to workers (retinue response)

**Figure 3:**
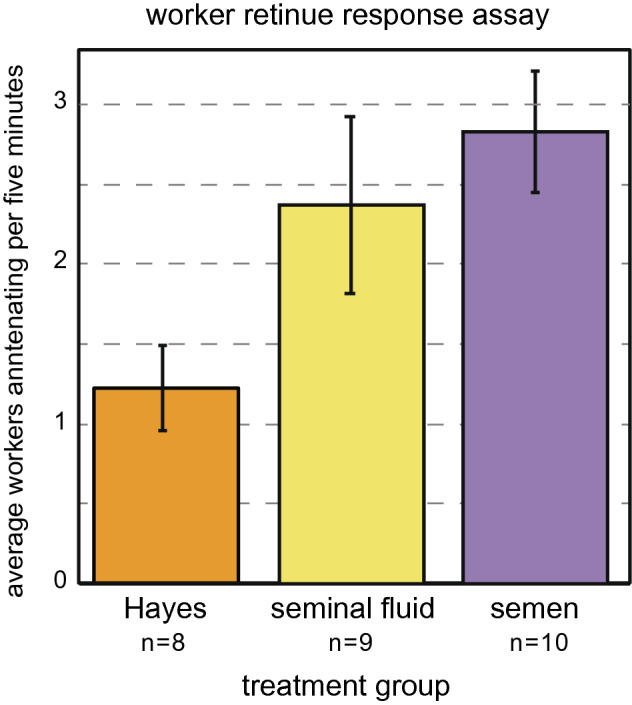
Worker retinue response assay. Workers were more attracted to both seminal fluid- and semen-injected queens compared to Hayes-injected queens (Pairwise t-tests; Bonferroni adj. p-values = 0.049, 0.019). Error bars represent standard error of the mean.

The number of workers forming a retinue around the queen is associated with the maturation of a queen’s pheromone profile and is a proxy of reproductive health^114^. SF and SE queens elicited greater worker retinue responses as compared to HS queens (Repeated measures ANOVA followed by post-hoc pairwise t-test; Bonferroni adj. p-values = 0.049, 0.019; Fig. 3, non-injected virgin control data is shown in Supplemental Fig. S3). There was no significant difference in retinue response between SF and SE queens (pairwise t-test; Bonferroni adj. p-value = 1).

### Ovary activation

Injections had no observable effect on ovary activation during the study period; there were no significant differences in ovary scores between any of the four treatment groups (Non-parametric Kruskal–Wallis test; df = 3, Chi-square value = 1.29, P = 0.73; Supplemental Fig. S4).

### Gene expression and pathway enrichment

After excluding sample outliers based on visual Multidimensional Scaling (MDS) plot assessment and DWV genome alignment rates (see methods), a total of 15 fat body and 11 brain RNAseq libraries were analyzed for differential expression using Limma. After filtering out genes with fewer than 20 reads in each sample, transcripts from 8,201 genes were detected in fat bodies and transcripts from 9,030 genes were detected in brains.

#### Differential expression in fat bodies

**Figure 4:**
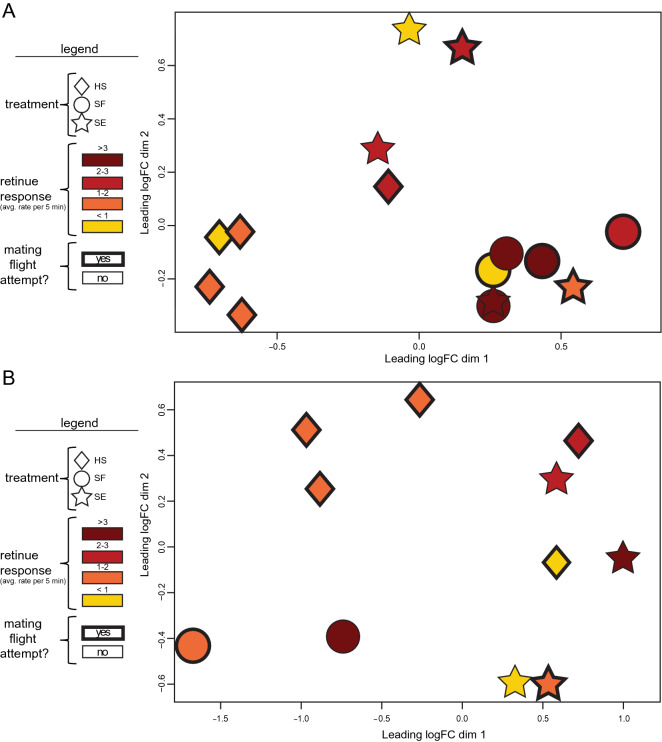
Multidimensional Scaling (MDS) plot of the top 500 expressed genes of (**A**) fat body samples and (**B**) brain samples generated with Limma in R. Distances between samples are approximations of typical (root-mean-square) log2-fold-change between the samples. Samples that are clustered closer to each other have less dissimilar expression profiles than samples that are located farther apart. Different shapes represent the different treatments: queens injected with Hayes Solution (HS), seminal fluid-injected queens (SF), and semen-injected queens (SE). Colors represent the average number of workers attenuating each queen per five minutes. Shapes with thick outlines represent queens that attempted a mating flight. (**A**) The expression profiles of fat body samples tended to cluster by treatment, and fat body samples from queens that were injected by semen or seminal fluid tended to cluster together. (**B**) The expression profiles of brain samples appeared to cluster by treatment except that SE queens were dispersed along the Y-axis.

The analyzed set included five HS, five SF, and five SE samples. SF and SE queens exhibited 131 and 342 DEGs, respectively, as compared to the HS queens (Table 1; Supplemental Tables S2–S3). However, there were no statistically significant DEGs between fat bodies from SF and SE queens. MDS plots overlaid with treatment information, worker retinue response data, and mating flight data were created to determine how similar the individual expression profiles of fat body samples were to each other and to assess if worker retinue response or mating flight behavior drove gene expression (Fig. 4A). Overall, fat body samples tended to cluster by treatment, except for samples from SE queens, which had more diverse expression profiles.

**Figure 5:**
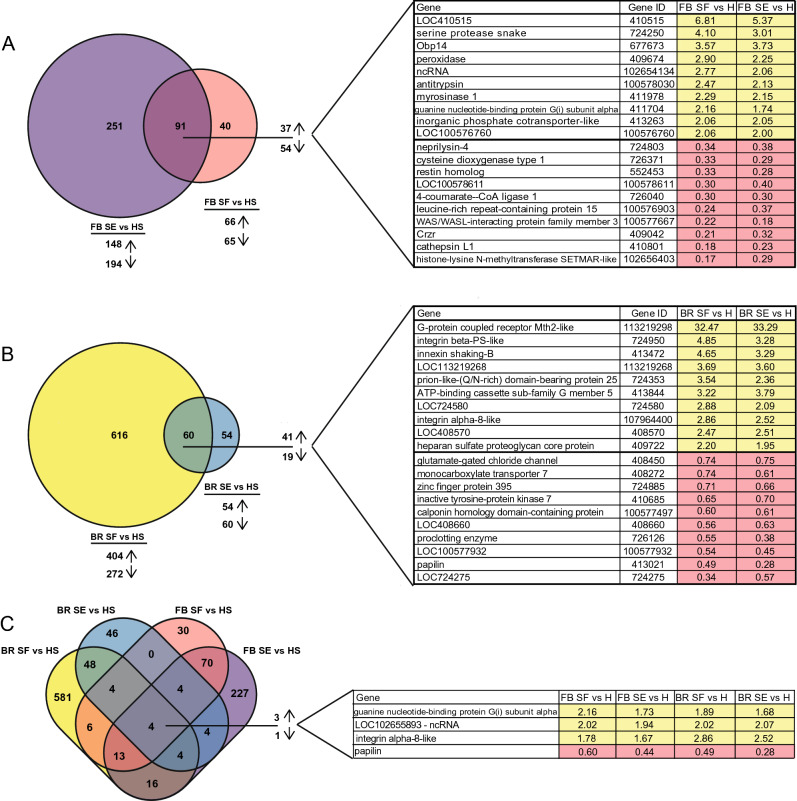
Venn diagram of differentially expressed genes (DEGs) in the brains and fat bodies of queens injected with semen or seminal fluid as compared to queens injected with Hayes solution. Numbers indicate number of DEGs and arrows represent number of DEGs that were upregulated and DEGs that were downregulated. (**A**) In the fat bodies, there were 131 DEGs in seminal fluid-injected queens (FB SF) as compared to Hayes-injected queens and there were 342 DEGs in queens injected with semen (FB SE) as compared to Hayes-injected queens (FB HS). There were 91 DEGs that were shared between the two pair-wise comparisons. The table displays the top ten upregulated DEGs and top ten downregulated DEGs with the greatest fold changes. (**B**) In the brains, there were 114 DEGs in queens injected with semen (BR SE) as compared to Hayes-injected queens and there were 676 DEGs in seminal fluid-injected queens (BR SF) as compared to Hayes-injected queens (BR HS). There were 60 DEGs that were shared between the two pair-wise comparisons. The table displays the top ten upregulated DEGs and top ten downregulated DEGs with the greatest fold changes. (**C**) Venn diagram analysis was performed to determine shared DEGs in the four pairwise comparisons: FB SF vs HS, FB SE vs HS, BR SF vs HS, and BR SE vs HS. There were four DEGs that were shared between the four pair-wise comparisons that exhibited the same direction in expression fold change. The table displays all shared DEGs and their respective fold changes in the four pair-wise comparisons.

To determine which DEGs were shared between SF and SE queens as compared to HS queens (Fig. 5A) we performed Venn diagram analysis. There were 91 shared DEGs, all of which shared the same directionality (Supplemental Tables S2, S3). Ten genes with the greatest increase in expression in SE and SF queens as compared to HS queens were *LOC410515* (410515), *serine protease snake* (724250), *odorant binding protein 14* (67767), *peroxidase* (409674), a non-coding RNA (ncRNA) (102654134), *antitrypsin* (100578030), *myrosinase 1* (411978), *guanine nucleotide-binding protein G(i) subunit alpha* (411704), *inorganic phosphate cotransporter-like* (413263), and *LOC100576760* (100576760) (Fig. 5A). The genes with the greatest decrease in expression in SE and SF queens as compared to HS queens were: *neprilysin-4* (724803), *cysteine dioxygenase type 1* (726371), *restin homolog* (552453), *LOC100578611* (100578611), *4-coumarate–CoA ligase 1* (726040), *leucine-rich repeat-containing protein 15* (100576903), *WAS/WASL-interacting protein family member 3* (100577667), *corozonin receptor* (*Crzr*) (409042), *cathepsin L1* (410801), and *histone-lysine N-methyltransferase SETMAR-like* (102656403) (Fig. 5A).

#### Differential expression in brains

The analyzed set included five HS, two SF, and four SE samples. SF and SE queens exhibited 676 and 114 DEGs, respectively, as compared to the HS queens (Table 1; Supplemental Tables S4, S5). MDS plots were generated to determine if individual expression profiles of brains samples clustered by treatment (Fig. 4B). Similar to the fat body samples, brain samples somewhat tended to cluster by treatment, except for samples from SE queens, which were spread farther along the Y-axis.

Venn diagram analysis on the brain sample subset was performed to determine which DEGs were shared between the brains of SF and SE queens as compared to HS queens (Fig. 5B). There were 60 shared DEGs, for which all shared the same directionality (Supplemental Tables S4–S5). The ten genes with the greatest increase in expression in SE and SF queens as compared to HS queens were *G-protein coupled receptor Mth2-like* (113219298), *integrin beta-PS-like* (724950), *innexin shaking-B* (413472), *LOC113219268* (113219268), *prion-like-(Q/N-rich) domain-bearing protein 25* (724353), *ATP-binding cassette sub-family G member 5* (413844), *LOC724580* (724580), *integrin alpha-8-like* (107964400), *LOC408570* (408570), and *heparan sulfate proteoglycan core protein* (409722). The ten genes with the greatest decrease in expression were *glutamate-gated chloride channel* (408450), *monocarboxylate transporter 7* (408,272), *zinc finger protein 395* (724,885), *inactive tyrosine-protein kinase 7* (410685), *calponin homology domain-containing protein* (100577497), LOC408660 (408660), *proclotting enzyme* (726126), *LOC100577932* (100577932), *papilin* (413021), *LOC724275* (724275).

Another Venn diagram analysis was performed in order to identify DEGs between SF and SE as compared to HS queens that were shared in both tissue types (Fig. 5C) (Supplemental Tables S2–S5). All four DEG lists shared four DEGs, which followed the same direction in expression change. Three of the shared DEGs were upregulated: *guanine nucleotide-binding protein G(i) subunit alpha* (411704), *LOC102655893*, which encodes a ncRNA (102655893), and *integrin alpha-8-like* (10796400). One of the shared DEGs was downregulated: *papilin* (413021).

#### Gene ontology analysis in fat bodies and brains

The *Drosophila melanogaster* orthologs of the DEGs from the following comparisons were analyzed via DAVID for gene ontology and functional enrichment analysis: fat body SF vs HS, fat body SE vs HS, brain SF vs HS, and brain SE vs HS (DEGs and their respective fly orthologs and GO annotations are available in Supplemental Tables S2–S5). However, no statistically significant GO clusters were identified in either of the fat body DEG lists or in the brain SE vs HS DEG list (modified Fisher’s exact test, threshold of p < 0.05 after BH correction). Several GO clusters were identified in the brain SF vs HS list, including GO:0002181 ~ cytoplasmic translation (23 genes, BH adj. p-value < 0.05), GO:0005576 ~ extracellular region (44 genes, BH adj. p-value < 0.005), GO:0044445 ~ cytosolic part (27 genes, BH adj. p-value < 0.005),

GO:0022626 ~ cytosolic ribosome (23 genes, BH adj. p-value < 0.005),

And GO:0000502 ~ proteasome complex (13 genes, BH adj. p-value < 0.05).

### qPCR validation of RNAseq results

Four genes (i.e., *serine protease snake*, *LOC408643*, *Antitrypsin*, and *LOC409674)* were upregulated in the fat bodies of SF and SE queens as compared to HS queens. The upregulation of these genes was validated using qPCR and the ΔΔC(t) method where *rpl8* was assayed as the housekeeping gene (Supplemental Fig. S5).

#### Transcriptome comparisons with prior work

**Figure 6:**
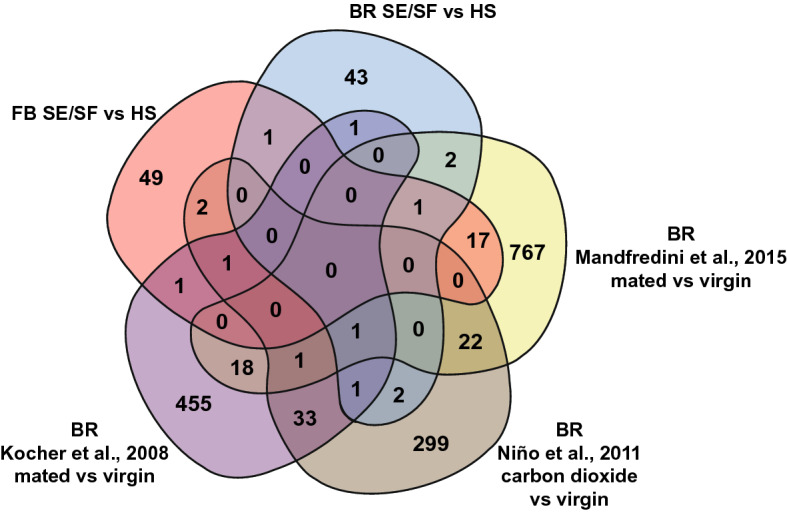
Venn diagram analysis of DEGs regulated in SF and SE queens as compared to HS queens and DEGs from previously published works. BR indicates that the tissue examined was brain and FB indicates that the tissue examined was fat body.

DEGs from our RNAseq analysis were compared to the differential expression results from prior published works examining transcriptional changes in the brains, ovaries, or fat bodies of honey bee queens that have undergone natural mating, instrumental insemination, and exposure to physical manipulation or CO_2_^37–42^. We compared all DEG lists based on their available *Apis mellifera* Amel_HAv3.1 BeeBase accession numbers (Supplemental Tables S6). 72 of the 91 shared SE and SF fat body DEGs had BeeBase annotations and 52 of the 60 shared brain DEGs had BeeBase accession numbers. Since visual Venn diagram analysis was limited to comparing five lists at a time, a diagram was made of the shared brain DEGs, shared fat body DEGs, and three DEG lists from previously published works that had the greatest number of shared DEGs (Fig. 6 and Supplemental Tables S6–S8)^37,38,40^. There were no DEGs that were shared amongst the five lists. Notably, there were 21 shared DEGs between the shared FB DEGs and mated vs virgin queens from the Manfredini et al.^37^ study (Table 2).

**Table 2:** DEGs shared between the fat bodies of SF and SE queens vs HS queens (this study) and the brains of mated vs virgin queens (Manfredini et al.^37^).

Shared genes with SF/SE vs HS in fatbodies		
BeeBase	Gene ID	Gene description
GB49363	412630	Scavenger receptor class B, type 5
GB48937	413575	Facilitated trehalose transporter Tret1-like
GB46223	677673	Odorant binding protein 14
GB44494	411657	Glucosinolate sulphatase
GB48269	100577569	Uncharacterized LOC100577569
GB46629	100577537	Uncharacterized LOC100577537
GB50889	551587	RNA-binding protein 38-like
GB40769	412458	Dehydrogenase/reductase SDR family member 11-like
GB40077	726040	Probable 4-coumarate–CoA ligase 3-like
GB50655	726371	Cysteine dioxygenase type 1-like
GB45034	726913	Fatty acid desaturase 1-like
GB46795	413021	Papilin-like
GB47668	409999	Lipase 3-like
GB55510	410801	Cathepsin J-like
GB54319	410052	Synaptotagmin 20
GB49336	552286	Acetyl-CoA carboxylase-like
GB44824	409042	Corazonin receptor
GB54315	724126	LOC724126

## Discussion

In this study, we examined the behavioral, physiological, and transcriptional responses of queens that were injected in the hemocoel with seminal fluid or semen in order to determine if components in seminal fluid alone contribute toward post-mating changes in honey bee queens. We found that injection with seminal fluid (SF) or semen (SE), containing both seminal fluid and sperm, reduces sexual receptivity and increases worker retinue response to queens as compared to queens injected with Hayes solution (HS) (Figs. 2 and 3). SF and SE queens also exhibited differential expression of more than a hundred genes in the fat body and brain as compared to HS queens (Table 1). However, we did not observe statistically significant changes in gene expression between the fat bodies of SF and SE queens, potentially because we missed the timeframe for observing these differences or because these changes are driven solely by seminal fluid components. Although we observed differences between SE/SF queens and HS queens, it is important to note that it has yet to be determined if seminal fluid components naturally exit the reproductive tract of honey bee queens, so the biological relevance of our hemocoel injection method in honey bees it is not yet known.

It is also important to remember that none of the queens in our study underwent ovary activation (Fig. 4), indicating that injection without CO_2_ sedation has no effect on ovary activation, or least not within the experimental timeframe. It also potentially indicates that seminal fluid or semen alone do not induce ovary activation, but future studies utilizing instrumental insemination without CO_2_ sedation will need to be performed in order to confirm this. However, our data corresponds with our earlier study in which greater ovary activation was not observed in queens inseminated with semen as compared to queens inseminated with saline solution, but greater insemination volumes resulted in greater ovary activation^41^. This suggests that ovary activation may be influenced more so by insemination volume or innervation of pressure sensors in the vaginal tract^41^.

We observed that mating flight attempts tended to be slightly lower and retinue response tended to be slightly higher (not significant) in SE queens as compared to SF queens. This suggests that while sperm could contribute to the reduction of sexual receptivity and modulation of pheromones resulting in greater attraction from workers, components in seminal fluid such as SFPs may have greater impact on both phenotypes associated with post-mating changes. Seminal fluid contains proteins, peptides, sugars, and lipids^114^. The role of seminal fluid and SFPs on female fertility and behavior and spermatozoa viability have been extensively examined in the model insect *Drosophila melanogaster* (*D. mel*) and less so in other insects, including crickets, ants, moths, and beetles^45–74,114^. The *D. mel* seminal fluid proteome contains specific proteins that reduce female sexual receptivity (i.e., sex peptide^86^), maintain spermatozoa viability (i.e., Acp29AB^115^), and promote uterine contractions (i.e., Acp36DE^116^) and ovulation (i.e., ovulin^117^). However, the honey bee genome does not encode homologs for any of these specific proteins^114^. Honey bee seminal fluid does maintain spermatozoa longevity, but the specific components in seminal fluid responsible have not been identified^58,118^. Interestingly, drone seminal fluid contains several odorant binding proteins and chemosensory protein 3^119,120^, which is also present in bee antennae and binds to fatty acids in order to influence behavior^121^. Odorant binding and chemosensory proteins may also aid in the solubilization and release of pheromones^122,123^. Thus, receipt of chemosensory protein 3 and odorant binding/chemosensing proteins from drone seminal fluid may induce changes in the queen brain that influence her sexual receptivity and pheromone production/release^119^. Similarly, lipids from drone seminal fluid may also influence queen post-mating changes, since reduced sexual receptivity in bumble bee queens is mediated by the receipt of fatty acids after mating^119,124,125^.

While drone seminal fluid may provide queens with odorant binding proteins and chemosensory proteins that affect sexual receptivity and pheromone production, our SE and SF queens exhibited increased expression of *odorant binding protein 14* (*Obp14*) and decreased expression of *chemosensory protein 1* (*Csp1*) as compared to HS queens. *Obp14* also exhibited increased expression in the brains of mated queens as compared to virgin queens^37^. Odorant binding protein 14 can be detected in disparate tissues throughout the honey bee body, including the brain^37^, spermatheca^126^, and fat body (this study). Since *Obp14* was upregulated in the fat body (this study), it is possible that it has been coopted by unrelated tissues^127^ to engage in non-chemosensing processes. Odorant binding proteins also play a role in humoral immunity against bacteria and yeast in vertebrates^128^. Likewise, honey bees infected with viruses^129^ and *Nosema apis*^130^ exhibit increased expression of *Obp14*. *Csp1* has also been regulated in the fat bodies of queens inseminated with 8 μl of either saline or semen compared to virgins^41^, upregulated in the brains of queens that were physically manipulated and exposed to CO_2_ as compared to virgin queens^40^, and, according to three individual studies, upregulated in the brains of mated queens as compared to virgin queens^37–39^ (Supplemental Table S6). It is interesting that *Csp1* was downregulated in the fat bodies of SE queens in our study whereas it has been upregulated in the brains of mated queens^37–39^. Pheromones produced in the queen’s paired mandibular glands are largely responsible for causing behavioral and physical responses that workers exhibit when headed by a mated queen: increased worker retinue response, reduced rearing of new queens, reduced swarming, and inhibited ovary activation^131–139^. The Dufour’s gland, located near the dorsal vaginal wall, also elicits retinue responses in workers and modulates pheromone composition based on mating status^34,140,141^. Perhaps, due to tissue localization, brain Csp1 production is essential for queen mandibular gland pheromone modulation release whereas fat body-localized Csp1 may regulate Dufour’s gland pheromone composition and production.

There are a few caveats to our comparative analyses between our RNAseq results and those of other transcriptional studies examining the role of different mating factors on queen post-mating changes^37–42^. Importantly, the queens from these studies had different genetic backgrounds, samples were collected at different times post-treatment and times of the year, the studies used different techniques to “inseminate” or naturally mate, and the majority of prior studies^38–41^ utilized microarrays, not RNAseq, to analyze gene expression with the exception of ^37,42^. That said, out of the 91 shared DEGs between the fat bodies of SF vs HS and SE vs HS (Fig. 5), several of them were also regulated in prior transcriptome studies. For example, *peroxidase* (LOC409674) was upregulated in fat bodies. The gene ontologies for the fruit fly homolog of peroxidase are oxidation–reduction process and response to oxidative stress (Supplemental Table S9). Similarly, queens exhibit increased expression of genes encoding for catalase, glutathione-S-transferase (GST), and superoxide dismutase 1 SOD1 in the spermatheca one year after mating, possibly as a mechanism to protect sperm that are stored in the spermatheca^142^. We observed *peroxidase* upregulation in the fat body, although it is unknown if peroxidases from the fat body would be utilized to preserve sperm in newly mated queens.

Similarly, *serine protease snake*, which was also upregulated in the fat bodies of SE and SF queens compared to HS queens, was also upregulated in the brains of queens exposed to CO_2_ and/or physical manipulation of the vaginal canal as compared to untreated virgin queens^40^, the fat bodies of queens inseminated with either 1 μl or 8 μl of semen or saline compared to untreated virgin queens^41^, and in two individual studies examining the brains of egg-laying queens or newly mated queens compared to virgin queens^38,39^. Serine protease snake plays a role in protein degradation, development, and immunity in honey bees and insects^143^. Interestingly, serine protease snake is a protein found in male ant sperm that likely plays a role in sperm competition and degradation of rival sperm^62,144^. Subsequently, serine protease snake proteins are then degraded by spermathecal secretions from the ant queen^144^. Antitrypsin (also known as serpin 4), which was also upregulated in the fat bodies of SE and SF queens and previous transcriptional studies^38–41^, is another protease with roles in immunity and potentially in sperm competition^143,145^. Proteases are an enriched class of genes that are upregulated in *Drosophila* females after mating and are highly enriched in the spermathecal proteome, thus they may function in sperm storage or regulation of proteolytic pathways^146^. This suggests that serine protease snake and antitrypsin, in the context of honey bee queen fat bodies, could play a role in regulating sperm competition.

*Drosophila melanogaster* seminal fluid sex peptide is responsible for regulating sexual receptivity and egg laying behavior in females^86^. Sex peptide binds to and interacts with a G-coupled protein receptor, sex peptide receptor^147^, found to be located in ppk + /fru- neurons associated with the female *D. mel* reproductive tract^147–149^. Although, *Apis mellifera* does not encode a homolog for sex peptide, it does encode a homolog (724,225) of sex peptide receptor, from which RNA transcripts were detected in the fat body and brain transcriptomes but were not differentially regulated in our study.

In *D. mel*, the sex peptide receptor requires guanine nucleotide-binding protein G(i) subunit alpha (Gα_i_) or guanine nucleotide-binding protein G(o) subunit alpha (Gα_o_) via the cAMP pathway for SP-induced activation^147^. In our study, *Gα*_*i*_ (411,704) was upregulated in the both fat bodies and brains of SE and SF queens as compared to HS queens (Fig. 5C). While Gα_i_ may have been engaging with multiple unrelated G-coupled protein receptors and signal transduction cascades, it is tempting to hypothesize that Gα_i_ plays a role in regulating sexual receptivity in queens. That said, *Gα*_*i*_ has not been observed to be regulated in queens in response to mating or mating stimuli in other studies, but sex peptide receptor was upregulated in mated queens and queens exposed to CO_2_ as compared to virgin queens^37^. Thus, the reduction of mating flight attempts/sexual receptivity in honey bee queens may only be partly due to activation of sex peptide receptor and its co-activators.

Along those same lines, *corazonin receptor* (*crzr*) was greatly downregulated in the fat bodies of SE and SF queens as compared to HS queens. While *crzr* is not well characterized in honey bees, the corazonin neuropeptide ligand and its receptor have been identified in insects, crustaceans, and ticks^150^. Corazonin contributes toward initiating the ecdysis behavioral sequence and suppresses silk production in moths^150^ and is predominantly expressed by workers in social insects^151^. *Harpegnathos* workers that convert into reproductive gamergates downregulate corazonin and corazonin inhibits vitellogenin expression and egg-laying in *Harpegnathos* and *Drosophila*^151^. This indicates that reduction in *crzr* expression in our experimental queens may have inhibited corazonin in preparation for ovary activation and subsequent egg-laying.

Several of the genes that were regulated in SE and SF queens as compared to HS queens play a role in photoreceptor-related activities in *D. mel*. For example, *fatty acid transport protein 4* (*FATP4*) and *cathepsin L1* were downregulated. *FATP4* is needed for photoreceptor neuron survival^152^ and cathepsins play a role in endocytosis-mediated retinal degeneration^153^. While these genes were not regulated in a prior study examining the loss of photoreception in mated honey bee queens^42^, the regulation of these genes in our study suggests their role in reducing queen phototaxis and sexual receptivity.

Many of the fat body and brain RNAseq libraries had high numbers of reads that aligned to the Deformed Wing Virus genome which may have confounded our differential gene expression results. However, considering that our results found significant differences in DWV alignment rates between virgin queens and injected queens but not amongst injected queens, we are confident in our results from comparing SE and SF to HS queens. DWV is considered ubiquitous in honey bee colonies^154^, so it is difficult to obtain DWV-free research samples.

## Conclusions and future directions

Our data indicate that injection of seminal fluid into the hemocoel induces several post-mating changes that are observed in naturally mated queens (i.e., reduced sexual receptivity, increased worker retinue response, and differential fat body and brain gene expression)^38,39^. Together with prior findings, our current results help further our understanding of mechanistic processes of honey bee queen mating and reproduction and provide a foundation for future studies. For example, this study provides potential queen gene targets (e.g., *carozonin*, *peroxidase*, *Gα*_*i*_) for dsRNA or siRNA gene knockdown in order to further examine their roles in shaping queen reproductive phenotype (e.g., egg-laying behavior and sperm viability in the spermatheca).

It will be important to determine if the non-protein portion of honey bee drone seminal fluid influences queen post-mating changes and to identify what specific metabolites are involved. One basic approach may involve fractionating seminal fluid into a protein fraction and a non-protein fraction as in Ram et al., 2005^155^ and testing their effects on queen post-mating changes. In two cricket species, *Teleogryllus commodus* and *Acheta domesticus*, seminal fluid-provided prostaglandins that reduce sexual receptivity and promote oviposition in recipient females^156,157^. It is not known if prostaglandins are present in drone seminal fluid or influence queen post-mating behavior, but they do play a role in honey bee immunity^156^. Additional studies using metabolomics or peptidomics approaches could be important towards elucidating how non-protein molecules in seminal fluid molecules may affect queen health and reproduction.

The proteome is the best characterized of the possible biomolecules in honey bee seminal fluid, and there is greater evidence for the role of proteins in female insect post-mating changes. The seminal fluid proteomes of different genetic lineages of bees exhibit different relative abundances and post-translational modifications of SFPs^158^. These naturally occurring differences in seminal fluid proteomes could be exploited to test their differential effects on queen reproductive phenotype. Furthermore, specific SFPs and their functions could be identified via fractionation of proteins (e.g., ion chromatography) and testing their individual effects^120^. In addition, advances in RNAi mediated gene knockdown^147^ honey bee CRISPR-Cas9 gene knockdown techniques^159^ will be important for identifying both drone and queen genes important for initiating post-mating changes and subsequent colony health.

While the proteins in the drone seminal fluid proteome have been identified, their target female tissues have not been identified. While our experimental queens were injected into the hemocoel, it has not yet been established if honey bee seminal fluid components exit the reproductive tract as they do in *D. mel*. Thus, in addition to determining the roles of specific SFPs on honey bee queen post-mating behavior and physiology, it will be important to determine the queen tissues, molecular targets, and receptors that interact with SFPs to induce these post-mating changes. Studies in *D. mel* have determined that SFPs transit from the reproductive tract into the hemolymph^160,161^ through the posterior vaginal wall of the female reproductive tract^162,163^. The specific female receptors to which *D. mel* SFPs bind are not well characterized, but Western blot analysis has determined that SFPs can traverse to the hemolymph and localize in the uterus, oviduct, sperm storage organ, ovaries, and even on mature oocytes and laid eggs^68,75,117,155,160,162^. Perhaps, through the development of honey bee SFP-specific antibodies, Western blot analysis can be used to determine queen SFP targets. This is indeed an exciting time in honey bee science and more targeted studies on queen reproduction are likely to contribute to reducing queen-related colony losses.

## Supplementary information

Supplementary figure 1

Supplementary figure 2

Supplementary figure 3

Supplementary figure 4

Supplementary figure 5

Supplementary tables

## Data Availability

The RNAseq datasets generated during the current study are available in the GEO repository (GSE145395). Behavioral datasets generated during the current study are available from the corresponding author on reasonable request.
