# Supplementary material for: Injection of seminal fluid into the hemocoel of honey bee queens (Apis mellifera) can stimulate post-mating changes
Source: Sci Rep. 2020 Jul 20;10:11990. doi: 10.1038/s41598-020-68437-w (PMC7371693; doi:10.1038/s41598-020-68437-w)
Supplement: Supplementary file 1 — Supplementary figure 1 [file 41598_2020_68437_MOESM1_ESM.pdf]

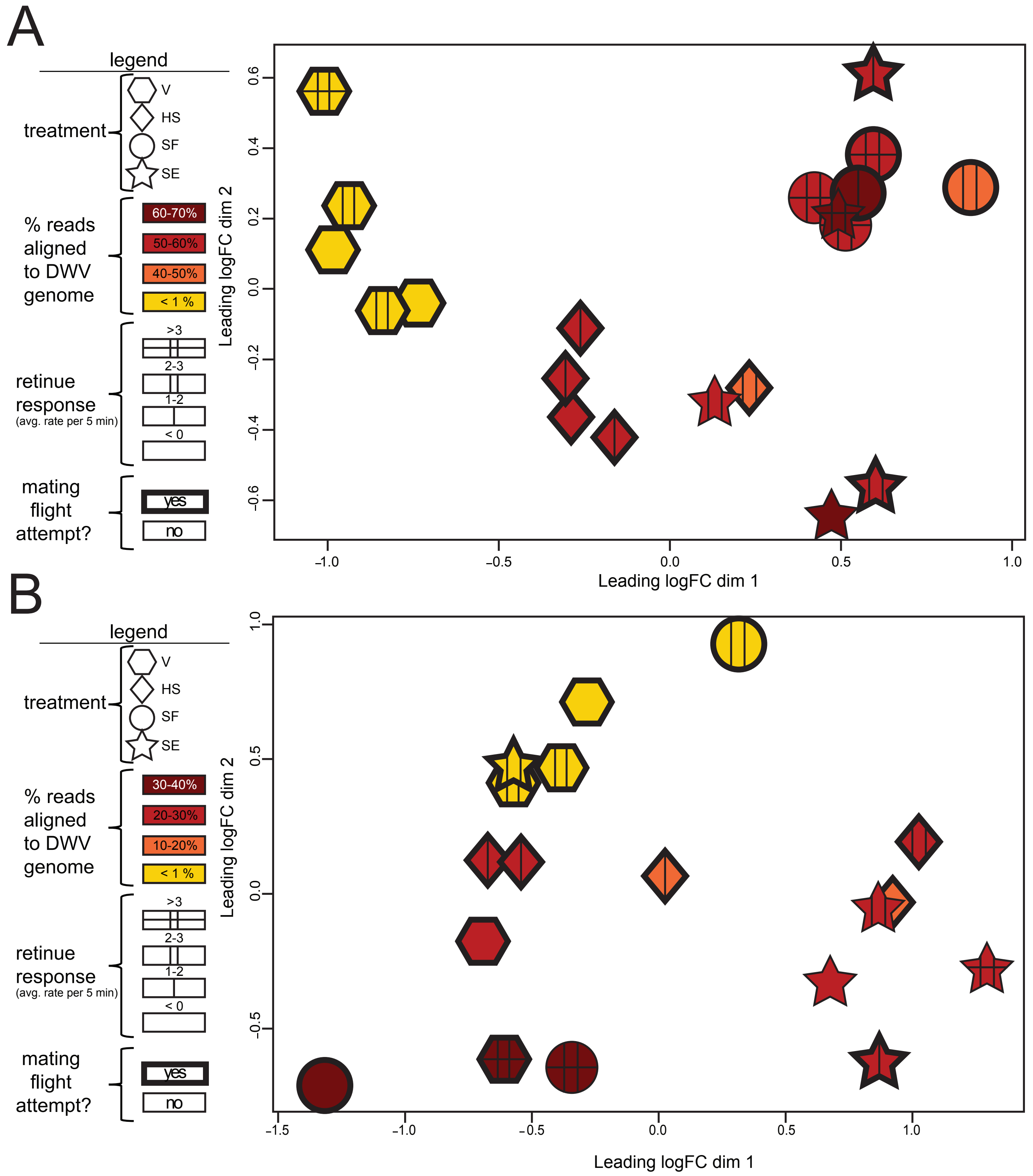

Supplemental Figure S1. Multidimensional Scaling (MDS) plot of the top 500 expressed genes of all (A) fat body samples and (B) brain samples generated using Limma in R. Distances between samples are approximations of typical (root-mean-square) log2-fold-change between the samples. Samples that are clustered closer to each other have less dissimilar expression profiles than samples that are located farther apart. Different shapes represent the different treatments: untreated virgin queens (V), queens injected with Hayes Solution (HS), seminal fluid-injected queens (SF), and semen-injected queens (SE). (Colors represent the percent of reads from each sample that aligned to the Deformed Wing virus genome. Line patterns within the shapes represent the average number of workers attenuating each queen per five minutes. Shapes with thick outlines represent queens that attempted a mating flight. (A) The expression profiles of fat body samples tended to cluster by treatment, and fat body samples from queens that were injected by semen or seminal fluid tended to cluster together. The % number of fat body reads aligning to the DWV genome were categorized into very high (>60% reads), high (50-60%), moderate (40-49%), and low (<1%). There were no samples that had DWV % alignment rates between 1-39%. (B) The expression profiles of brain samples appeared to cluster by percent of reads aligning to the DWV genome. The % number of fat body reads aligning to the DWV genome were categorized into very high (>30% reads), high (20-29%), moderate (0-19%), and low (<1%). When excluding samples with low rates of DWV genome alignment (>1%), samples appeared to cluster more so by treatment along the Y-axis, so differential expression analysis with Limma excluded this subset of samples.

Injection of seminal fluid into the hemocoels of honey bee queens (*Apis mellifera*) can stimulate post-mating changes

W. Cameron Jasper[1†], Laura M. Brutscher[1†], Christina M. Grozinger[2] and Elina L. Niño[1]*

[1] Department of Entomology and Nematology, University of California Davis, One Shields Ave, Davis, CA 95616, USA

[2] Department of Entomology, Center for Pollinator Research, Huck Institutes of the Life Sciences, Pennsylvania State University, University Park, 16802, PA, USA

[†] Co-first authors

* Corresponding author
Address: Department of Entomology and Nematology, University of California, 1 Shields Avenue, Davis, California, 95616
Telephone: 530-500-2747
Fax: 530-752-1537
Email: elnino@ucdavis.edu
