# Supplementary material for: Injection of seminal fluid into the hemocoel of honey bee queens (Apis mellifera) can stimulate post-mating changes
Source: Sci Rep. 2020 Jul 20;10:11990. doi: 10.1038/s41598-020-68437-w (PMC7371693; doi:10.1038/s41598-020-68437-w)
Supplement: Supplementary file 2 — Supplementary figure 2 [file 41598_2020_68437_MOESM2_ESM.pdf]

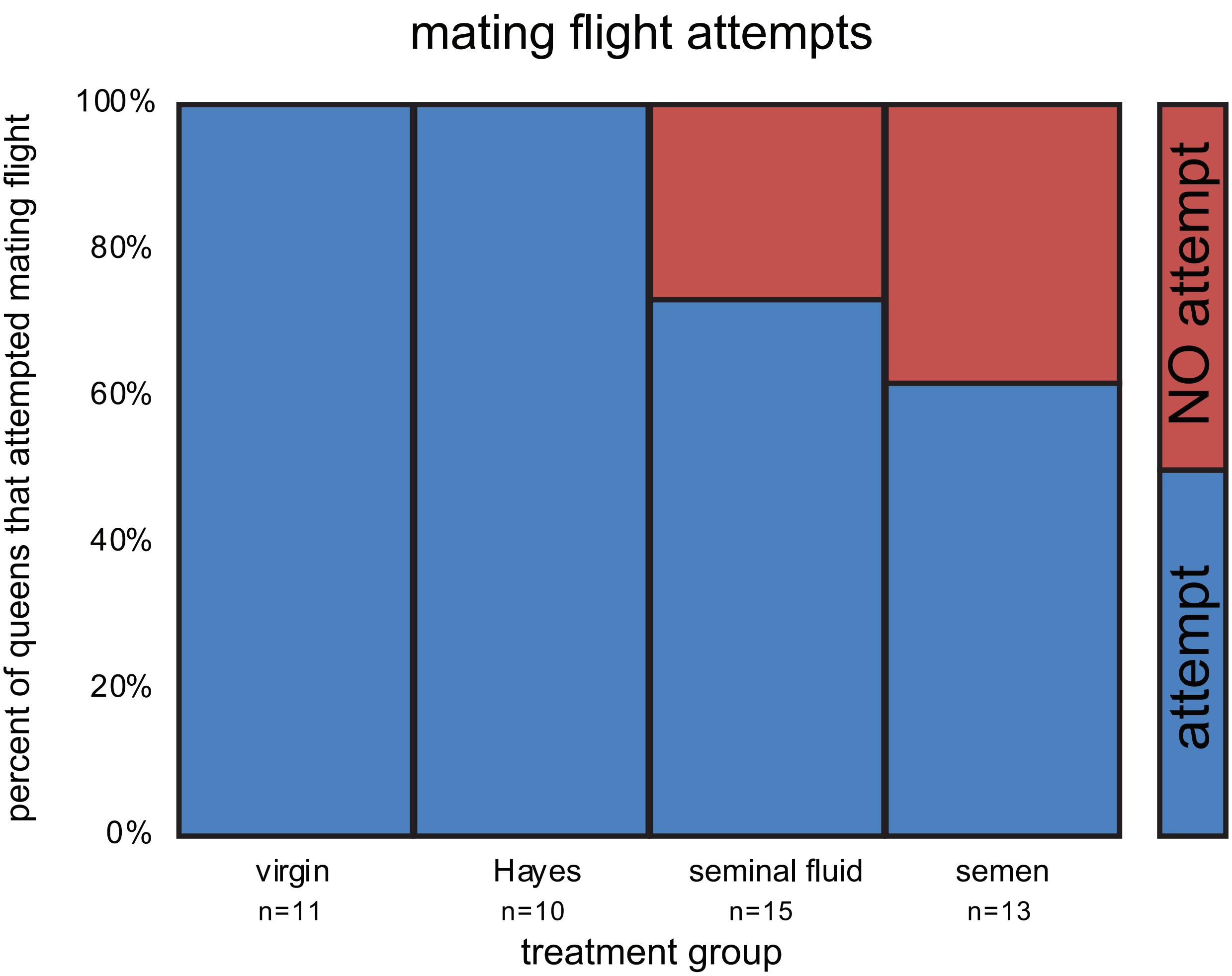

Supplemental Figure S2. Queen Mating Flight Attempts (sexual receptivity). Queens injected with semen or seminal fluid (grouped) have reduced sexual receptivity compared to virgin queens and queens injected with Hayes (grouped) (chi-square (Pearson) = 8 $\chi 2$= 19.61, df=1, P = 9.486e-06). Blue represents queens that attempted a mating flight and red represents queens that did not attempt a mating flight.

Injection of seminal fluid into the hemocoels of honey bee queens (*Apis mellifera*) can stimulate post-mating changes

W. Cameron Jasper[1†], Laura M. Brutscher[1†], Christina M. Grozinger[2] and Elina L. Niño[1]*

[1] Department of Entomology and Nematology, University of California Davis, One Shields Ave, Davis, CA 95616, USA

[2] Department of Entomology, Center for Pollinator Research, Huck Institutes of the Life Sciences, Pennsylvania State University, University Park, 16802, PA, USA

[†] Co-first authors

* Corresponding author
Address: Department of Entomology and Nematology, University of California, 1 Shields Avenue, Davis, California, 95616
Telephone: 530-500-2747
Fax: 530-752-1537
Email: elnino@ucdavis.edu
