# Supplementary material for: Injection of seminal fluid into the hemocoel of honey bee queens (Apis mellifera) can stimulate post-mating changes
Source: Sci Rep. 2020 Jul 20;10:11990. doi: 10.1038/s41598-020-68437-w (PMC7371693; doi:10.1038/s41598-020-68437-w)
Supplement: Supplementary file 3 — Supplementary figure 3 [file 41598_2020_68437_MOESM3_ESM.pdf]

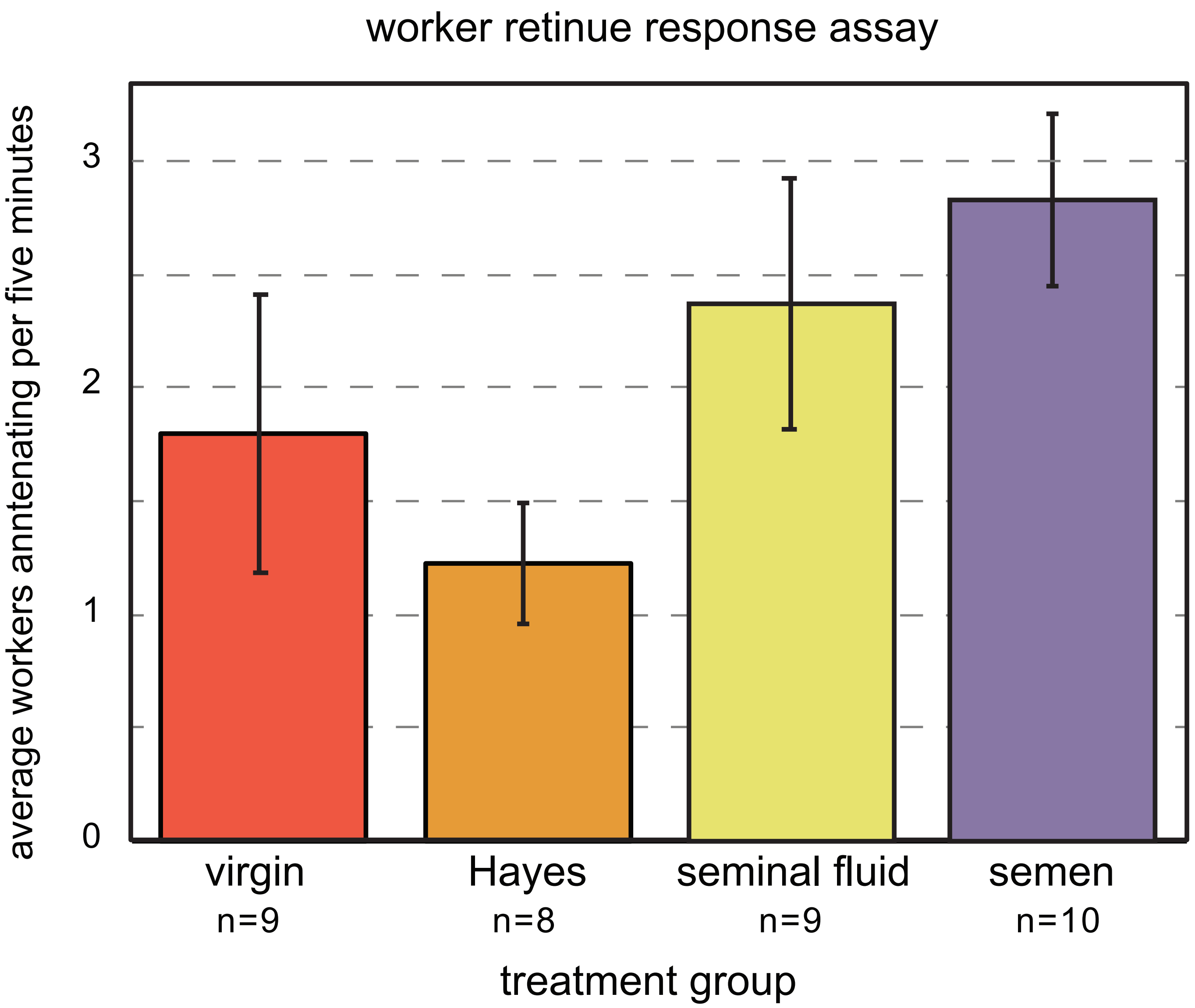

Supplemental Figure S3. Worker retinue response assay. Workers tended to be more attracted to both seminal fluid- and semen-injected queens than Hayes-injected queens, but this was not significant for SF queens (Repeated measures ANOVA followed by post-hoc Pairwise t-tests; Bonferroni adj. p-values=0.106, 0.042). However, upon excluding virgins from analysis, there is a significant difference between SF and SE queens as compared to HS queens (Repeated measures ANOVA followed by post-hoc Pairwise t-tests; Bonferroni adj. p-values=0.049, 0.019). Error bars represent standard error of the mean.

Injection of seminal fluid into the hemocoels of honey bee queens (*Apis mellifera*) can stimulate post-mating changes

W. Cameron Jasper[1†], Laura M. Brutscher[1†], Christina M. Grozinger[2] and Elina L. Niño[1*]

[1] Department of Entomology and Nematology, University of California Davis, One Shields Ave, Davis, CA 95616, USA

[2] Department of Entomology, Center for Pollinator Research, Huck Institutes of the Life Sciences, Pennsylvania State University, University Park, 16802, PA, USA

[†] Co-first authors

* Corresponding author
Address: Department of Entomology and Nematology, University of California, 1 Shields Avenue, Davis, California, 95616
Telephone: 530-500-2747
Fax: 530-752-1537
Email: elnino@ucdavis.edu
