# Supplementary material for: Injection of seminal fluid into the hemocoel of honey bee queens (Apis mellifera) can stimulate post-mating changes
Source: Sci Rep. 2020 Jul 20;10:11990. doi: 10.1038/s41598-020-68437-w (PMC7371693; doi:10.1038/s41598-020-68437-w)
Supplement: Supplementary file 4 — Supplementary figure 4 [file 41598_2020_68437_MOESM4_ESM.pdf]

Injection of seminal fluid into the hemocoels of honey bee queens (*Apis mellifera*) can stimulate post-mating changes

W. Cameron Jasper[1†], Laura M. Brutscher[1†], Christina M. Grozinger[2] and Elina L. Niño[1]*

[1] Department of Entomology and Nematology, University of California Davis, One Shields Ave, Davis, CA 95616, USA

[2] Department of Entomology, Center for Pollinator Research, Huck Institutes of the Life Sciences, Pennsylvania State University, University Park, 16802, PA, USA

[†] Co-first authors

* Corresponding author
Address: Department of Entomology and Nematology, University of California, 1 Shields Avenue, Davis, California, 95616
Telephone: 530-500-2747
Fax: 530-752-1537
Email: elnino@ucdavis.edu

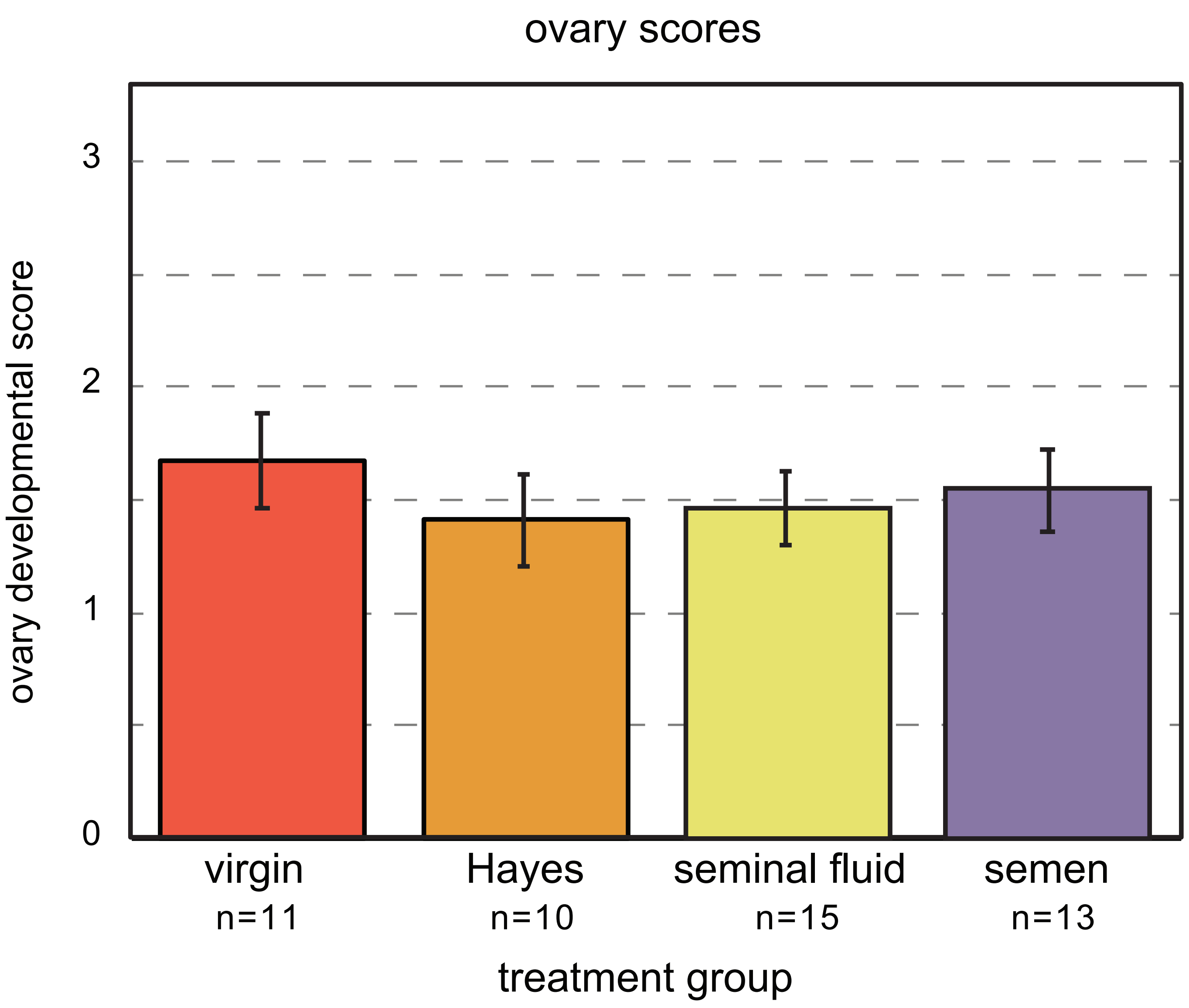

Supplemental Figure S4. Ovary developmental scores of queens injected with Hayes solution, seminal fluid, or semen and queens that were not treated (virgin). Ovaries were dissected and assessed for no development (score=1), thickening of ovarioles (score=2), presence of developing eggs (score=3), and presence of fully developed eggs (score=3). Overall, there was no statistical difference in ovary scores amongst the four treatment groups (Non-parametric Kruskal-Wallis test; df=3, Chi-square value=1.29, P=0.73).
