# Supplementary material for: Injection of seminal fluid into the hemocoel of honey bee queens (Apis mellifera) can stimulate post-mating changes
Source: Sci Rep. 2020 Jul 20;10:11990. doi: 10.1038/s41598-020-68437-w (PMC7371693; doi:10.1038/s41598-020-68437-w)
Supplement: Supplementary file 5 — Supplementary figure 5 [file 41598_2020_68437_MOESM5_ESM.pdf]

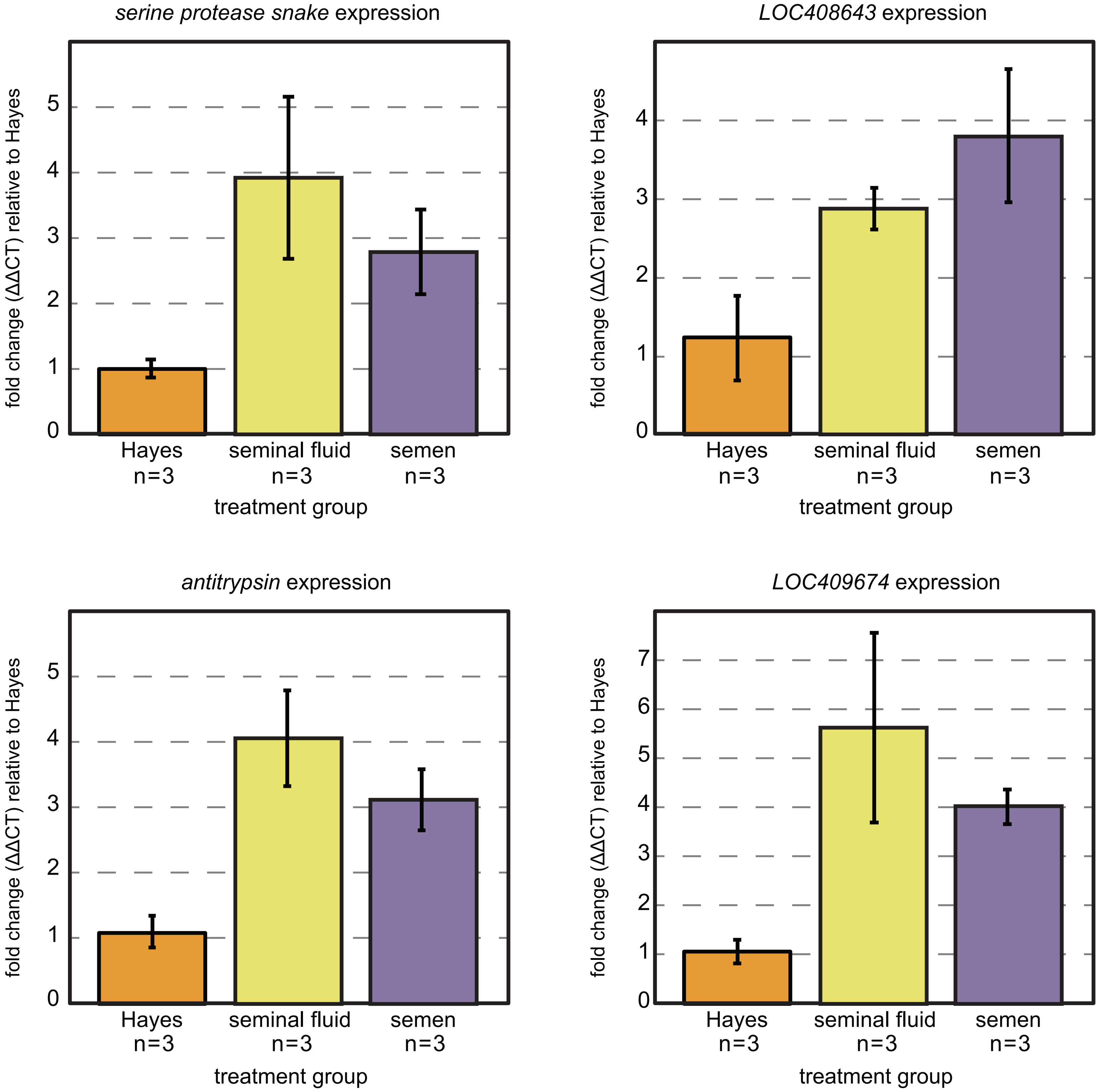

Supplemental Figure S5. qPCR validation of four genes that were determined to be differentially regulated via RNAseq. Based on RNAseq analysis of queens that were injected with Hayes solution (control), seminal fluid, or semen, *serine protease snake*, *LOC408643*, *antitrypsin*, and *LOC409674* were upregulated in both seminal fluid and semen-injected queens as compared in Hayes-injected queens. qPCR was then used to further validate these results by designing primers against the four genes and normalizing their expression against the housekeeping gene *Rpl8* (ΔCT) and then normalizing all ΔCTs against the average Hayes ΔCT using the ΔΔCT method. Bars represent standard error of the mean.

Injection of seminal fluid into the hemocoels of honey bee queens (*Apis mellifera*) can stimulate post-mating changes

W. Cameron Jasper[1†], Laura M. Brutscher[1†], Christina M. Grozinger[2] and Elina L. Niño[1]*

[1] Department of Entomology and Nematology, University of California Davis, One Shields Ave, Davis, CA 95616, USA

[2] Department of Entomology, Center for Pollinator Research, Huck Institutes of the Life Sciences, Pennsylvania State University, University Park, 16802, PA, USA

[†] Co-first authors

* Corresponding author
Address: Department of Entomology and Nematology, University of California, 1 Shields Avenue, Davis, California, 95616
Telephone: 530-500-2747
Fax: 530-752-1537
Email: elnino@ucdavis.edu
